# Interactions between Self-Perceived Weight Status and Lifestyle Behaviors and Their Associations with Childhood Obesity: Results from the Childhood Obesity Study in China Mega-Cities

**DOI:** 10.3390/ijerph19169921

**Published:** 2022-08-11

**Authors:** Liwang Gao, Jiang Zhu, Liang Wang, Li Ming Wen, Zhuo Chen, Bingtong Zhao, Weidong Wang, Youfa Wang

**Affiliations:** 1 Center for Non-Communicable Disease Management, Beijing Children’s Hospital, Capital Medical University, National Center for Children’s Health, Beijing 100045, China; 2 Global Health Institute, School of Public Health, Xi’an Jiaotong University Health Science Center, Xi’an 710061, China; 3 Department of Public Health, Robbins College of Health and Human Sciences, Baylor University, Waco, TX 76798, USA; 4 School of Public Health, Faculty of Medicine and Health, University of Sydney, Sydney 2006, Australia; 5 Department of Health Policy and Management, College of Public Health, University of Georgia, Athens, GA 30602, USA; 6 School of Economics, Faculty of Humanities and Social Sciences, University of Nottingham Ningbo, Ningbo 315199, China; 7 Department of Sociology, School of Sociology and Population Studies, Renmin University of China, Beijing 100034, China

**Keywords:** overweight and obesity, weight self-perception, behavioral factors, adolescents, China

## Abstract

This study investigated the interactions between self-perceived weight status and lifestyle behaviors, and their associations with childhood obesity among school children. **Methods**: Cross-sectional study data from a nationwide sample of 3258 participants (aged 8–15 years old) during 2015–2017 were used. Self-perceived weight status and lifestyle factors (dietary intake and physical activity) were assessed using self-administered questionnaires. Multivariable mixed-effects models tested the effect of interactions between weight self-perception and behavioral factors on overweight and obesity (ow/ob). **Results**: Overall ow/ob prevalence based on BMI was 30.9% (38.5% for boys, 23.0% for girls). Based on self-perceived weight status, ow/ob prevalence was 37.7% (35.8% for boys, 39.7% for girls). 41.2% of boys and 25.9% of girls underestimated their actual weight status. The interaction between self-perceived weight status and meat consumption was associated with ow/ob in boys, while the interaction between self-assessed weight status and protein foods and sedentary lifestyle were associated with ow/ob in girls. The attributable proportions of these three factors were 39.8%, 48.2%, and 34.6%, respectively. **Conclusions**: The self-perceived weight status was different from their actual weight status in children. The interactions between self-perceived weight status and lifestyle behaviors were associated with ow/ob. Health promotion programs that empower children to have appropriate self-awareness of weight status, eating, and physical activity behaviors need to be developed and implemented.

## 1. Introduction

With the rapid economic development and the enormous changes in people’s way of life, the prevalence of overweight and obesity in China has been showing a rapid growth trend [1]. A nationally representative report shows that more than half of adults in China were overweight or obese, and nearly 20% of school-age children and 10% of preschool children were overweight or obese in 2021 [2]. The epidemic of obesity has not only incurred heavy disease burdens and considerable economic losses but also affected the physical fitness and quality of life of the population [3,4,5]. Therefore, it is particularly important to understand the risk factors associated with childhood obesity and to take preventive and control measures [6,7].

Obesity is affected by many factors, such as psychological issues, behaviors, and body dissatisfaction [8,9]. Previous studies have shown that adverse lifestyle factors (such as high-energy food intake, reduced physical activity, and fast-food diets) are independent risk factors for childhood obesity, and the findings from lifestyle behavioral studies can help with advocating for childhood obesity interventions [10,11,12]. Misperception of body weight, defined as the mismatch between an individual’s measured weight status based on body mass index (BMI) and his/her perceived weight status, was also reported as a risk factor associated with obesity [13]. Our previous study showed that 49.0% of children underestimated their weight status, one-third of African–American adolescents did not correctly classify their weight status [14,15], and perceiving own’s body size as normal decreases the odds of weight-losing behavior [16].

Furthermore, there are also clear links between weight misperception and life behaviors: the direct association between underestimation and ultra-processed and sweet foods indicated that weight misperception was related to unhealthy eating habits in adolescents [17]. However, previous studies, including ours, have examined the association of weight perception or life behavior with weight status, respectively. No studies have discussed the interaction of weight misunderstandings and life behaviors on obesity.

To fill these knowledge gaps, this study aimed to investigate the differences between self-perceived weight status and measured weight status based on BMI, as well as the interactions between self-perceived weight status and lifestyle behaviors (i.e., eating and physical activity), and their associations with childhood obesity.

## 2. Materials and Methods

### 2.1. Study Design and Study Participants

This study conducted a cross-sectional analysis using data extracted from the Childhood Obesity Study in China Mega-Cities (COCM) project, a US National Institutes of Health-funded longitudinal study that aimed to examine the etiology of childhood obesity and chronic diseases in China, especially in five major cities (Beijing, Shanghai, Xi’an, Nanjing, and Chengdu). Detailed information about the COCM project has been reported elsewhere [18]. The study data was approved by the Ethical Committees of the State University of New York at Buffalo, Ball State University, and Xi’an Jiaotong University in China. Written informed consent was obtained from parents and children aged 8–15 years old.

For this study, we used data collected from 2015–2017 including weight status, lifestyle behaviors of children, and their family demographic characteristics. The sample size from the China Mega-Cities (COCM) project was 3369. For this analysis, we used the latest available observations, collected during 2015–2017, of 3258 students who participated in the surveys. Children with missing data on age, gender, weight, and height measurements were excluded from the analysis (*n* = 110).

### 2.2. Assessment and Measures

BMI and child weight status. Height was measured by a Seca 213 Portable Stadiometer Height-Rod with a precision of 0.1 cm; body weight was measured by a Seca 877 electronic flat scale with a precision of 0.1 kg. Subjects were measured by trained research staff using a unified model and the specified instruments in a quiet classroom. Overweight and obesity was defined according to the age- and gender-specific BMI cutoffs issued by the National Health Commission of the People’s Republic of China (Table A1 in Appendix A) [19].

Self-perceived weight status. This was defined by the question: “What do you think about your own body weight?” There were five options that ranged from “very thin” to “very obese.” Self-perceived weight status was classified into the dichotomous variables of overweight and non-overweight when we evaluated and analyzed the interactions.

Lifestyle behaviors. Information on lifestyle factors (dietary intake and physical activity) was collected through self-administered questionnaires, which were adapted from the questionnaires used in previous research projects by our team and proved to be reliable and valid through actual investigations [20]. Dietary intake was assessed by a set of questions which included the frequency of consumption of livestock meat, poultry, plain milk, dairy products, and soybeans and soy products. The dietary intake frequency of each item was categorized into two groups, namely skippers (those eating from that food category < 3 days/week) and non-skippers (those eating from the category 3–7 days/week). Physical activity was recorded as the number of days per week in which subjects spent at least 30 min/day participating in extracurricular physical activities such as cycling, roller-skating, running, swimming, dancing, team sports, tennis, etc. Physical activity intensity was divided into two categories, high or moderate (3–7 days/week) and low (<3 days/week). Sedentary behavior was determined by the time spent per day in a week either watching TV/videos/movies or going online/using a computer. It was classified into two categories: high or moderate (3–7 days/week) and low (<3 days/week). The Cronbach’s α of this scale was 0.87.

Parental and household characteristics. To consider the influence of family and household characteristics on the associations between children’s weight status and body image, we used parents’ BMI, highest parental education (up to primary school, middle school, vocational/college/higher degree[s]), and family home ownership (rent or share residency with relatives, own apartment, own house) in our analysis, which were obtained from the parent questionnaire filled out by the students’ parents.

### 2.3. Statistical Analysis

First, we described the distributions of children’s actual weight status and self-perceived weight status by their socio-demographics, and identified discrepancies between self-perceived weight status and actual weight status. A chi-square test was used to compare the differences in the categorical variables between boys and girls. One-way analysis of variance (ANOVA) was used for continuous variables.

For the lifestyle behaviors, we used factor analysis methods to extract common factors from the broad range of behaviors, and then studied the associations between obesity and interactions between these common behaviors and body image. The Kaiser–Meyer–Olkin (KMO) method and the Bartlett spherical test were used to confirm whether the data were suitable for factor analysis. By using the principal component method for exploratory factor analysis, the initial factors were extracted, and then rotated by the varimax orthogonal rotation method to obtain a more explicit common factor and to calculate the factor score.

Finally, the analysis of the interactions between lifestyle behavior and body image was performed using the Cochran–Mantel–Haenszel hierarchical analysis. A mixed-effects logistic model using the melogit command in Stata was used to assess the associations between ow/ob and the interactions of lifestyle behaviors and body image, and covariates were gender and age. We used Andersson’s Excel to calculate additive interaction [21].

Data analyses were conducted using Excel and Stata software version 15 (StataCorp, College Station, TX, USA). The effect size was presented as beta coefficients with a 95% confidence interval (CI) and the regression coefficient with a 95% CI. Statistical significance was set at *p* < 0.05.

## 3. Results

### 3.1. Study Participants’ Characteristics and Body Image

Table 1 shows the sample characteristics of study participants in the COCM survey. Of the 3258 children, the ratio of boys to girls was close to 1:1. The mean age was 12.2 (SD = 2.0) years old, and there was no statistically significant difference in age between boys and girls. Compared with girls, boys had higher BMI (*p* < 0.001), and higher consumption of livestock meat, poultry, plain milk, and dairy products (*p* < 0.05). Boys were also more likely to watch TV or movies than girls (*p* < 0.001).

**Figure 1:**
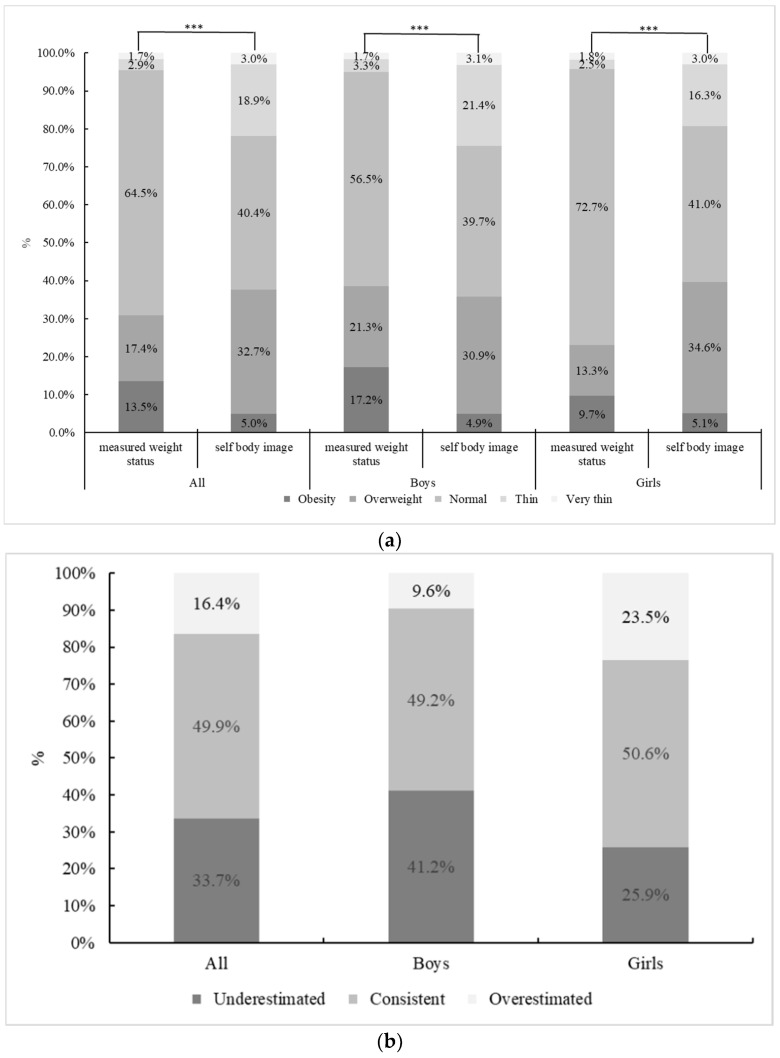
Data of self-perceived weight status (%) and child weight status (%) in 2015–2017. (**a**) Child measured weight status (based on BMI) and self-perceived weight status. (**b**) Sex differences in self-perceived weight status vs. measured weight status (based on BMI). Chi-square test was used to compare gender difference. *** *p* < 0.001.

As shown in Figure 1, 30.9% of children were obese. There was a statistically significant difference in weight status between boys and girls. Girls had a higher prevalence of ow/ob than boys (38.5% vs. 23.0%). Only 49.2% of boys and 50.9% of girls had a self-perceived weight status consistent with their measured weight status. Furthermore, 41.2% of boys and 25.9% of girls underestimated their actual weight status.

### 3.2. Factor Analysis: Eating and Physical Activity Behaviors

As listed in Table 2, exploratory factor analysis studied eight factors potentially associated with ow/ob, including consumption of livestock meat, poultry meat, plain milk, dairy products, and soybeans and soy products, as well as activities such as watching TV/videos/movies, going online/using a computer, and extracurricular physical activity. The KMO value of this study data was 0.711, and the Bartlett spherical test had a value of 2514.844 (*p* < 0.001), so these items were suitable for factor analysis. Upon extraction of the principal components, a total of four were found, and all four were common factors with a characteristic root >1. Data with factor loads <0.65 were eliminated. The cumulative contribution rate of variance was 72.19%. Factor 1 was mainly dominated by plain milk, dairy products, and soybeans and soy products and was named “protein foods”. Factor 2 was mainly dominated by meat and was named “meat foods”. Factor 3 was mainly dominated by watching TV and using computers and game consoles and was named “sedentary behaviors”. Factor 4 was mainly dominated by extracurricular activities and was named “extracurricular physical activity”.

### 3.3. Association between Obesity and Interaction of Self-Perceived Weight Status and Lifestyle Behavior Factors

Self-perceived weight status was defined as a dichotomous variable (non-overweight vs. overweight). The four factors were also divided into two categorical variables by the median percentile (P50) of their own factor scores. The association between body status and the interactions of body image and the four factors were analyzed separately using a mixed-effect model (Table 3). The results showed that the attributable proportion (AP) between self-perceived weight status and protein foods, meat foods, sedentary behaviors, and extracurricular physical activity in children was 26.2%, 21.8%, 19.1%, and 15.7%, respectively. The interactions were also estimated in different genders. In the calculation of AP, four additive interactions between self-perceived weight status and factors were statistically significant (Table A2). They were Factor 1, Factor 1 in girls, Factor 2 in boys, and Factor 3 in girls. The AP of these four factors was 26.2%, 48.2%, 39.8%, and 34.6%, respectively.

## 4. Discussion

Our data suggest that Chinese children demonstrate a large incongruence between their weight-status assessment and actual weight status. Only half the Chinese children in this study had an accurate body image. About 33.7% underestimated their true weight status, and 16.4% overestimated their weight status. These results showing misperception are higher compared with previous studies exploring the differences between BMI and body weight perception among children and adolescents [22], but the results are the same insofar as girls tend to perceive themselves as overweight more than boys do [23,24]. Boys were more likely than girls to underestimate their weight and preferred to select a larger ideal body shape, which may be influenced by peers, mass media, parents, and other sociocultural factors that promote slimness for girls and muscularity for boys [25,26], even though such social pressures may be unreasonable or unhealthy [27].

Consistent with previous research, our results also indicated that children who perceived themselves as being overweight had a higher risk of obesity than those with non-overweight. Both weight misperception and poor body image may have negative psychological and psychosocial effects (e.g., low self-esteem, anxiety, depression, isolation, discrimination, and family conflicts), which had a stronger association with binge eating for boys and girls [28]. People engaged in frequent self-evaluations (including body checking) comparing themselves to those who they believe have more desirable sociocultural traits and tend to be involved in behaviors aimed to achieve those desired characteristics [29]. This highlighted that proper education of weight cognition in children could contribute to the prevention and control of obesity.

Furthermore, we found that a high frequency of soy and dairy intake reduces obesity risk. This is consistent with the conclusion of the review study that proteins play an important role in reducing the risk of metabolic syndrome, diabetes management, cancer prevention and weight management [30]. The mechanism may be that soy products and dairy products are rich in high-quality protein, and the non-essential amino acids in these foods can reduce insulin resistance; some studies have found that proteins derived from soybean products can enhance fat oxidation and inhibit fat formation by antagonizing lipogenic enzymes [31,32].

Using exploratory factor analysis and interactive logical regression additive models, we also found that consumption of protein foods and meat, as well as sedentary behaviors, may affect the relationship between body image and obesity in Chinese school-age children. Possible reason is that children’s self-perceived weight status changes their lifestyle behaviors, which in turn affects weight status [16], as research showed that people’s body shape and/or image play an important role in how they behave [33].

School is an important institution to promote the healthy development of children and adolescents, and the present results showed that there is a mismatch between BMI and body weight perception. Therefore, schools and parents should take targeted measures to help teenagers improve their weight management skills and raise their health awareness. First, school leaders, teachers, and parents should be trained to improve students’ health awareness, help them improve or strengthen their health-related behaviors, and create a positive environment for them to grow. Second, due to the pressures of school admission in China, many extracurricular physical activities have been canceled. To address the growing obesity problem and mitigate the future societal costs of ow/ob in tomorrow’s adults, schools today need to sponsor more extracurricular activities to encourage the habit of physical exercise. In addition, schools should teach students the principles of good lifelong health. These measures will improve the accuracy of students’ body image, their ability to manage their weight appropriately, and their adoption of reasonable eating habits.

In our study, some limitations must be noted. First, the research was a cross-sectional study, so causality could not be determined. Second, the information on dietary and lifestyle behavior factors in this study were collected by questionnaire, but accurate information on energy intake and energy consumption could not be obtained. Third, although this study found that the interaction between body image and lifestyle behavior was independent, after controlling for the effects of covariates such as sex, age, and parental and household characteristics, it was still uncertain whether this interaction was due to the residual confounding effects of other unmeasured factors, such as mental health.

While we acknowledge these limitations, our survey has the following strengths. First, this study used a representative sample of children from major Chinese cities that have experienced rapid socio-economic changes over the past three decades. These transitions have resulted in many dramatic changes in the social environments and people’s lifestyles, which have led to an increase in obesity and other health problems. The results could provide useful information for policymakers in making informed decisions to promote the healthy growth of children and adolescents. Second, we calculated actual weight status by measuring height and weight, which is necessary for calculating BMI, rather than relying on self-reported height and weight. Third, we emphasized the importance of body weight perception and analyzed the interactions between self-perceived weight status and lifestyle behaviors and their association with childhood obesity. Misperception is harmful to the health and normal growth of young people, and it works with lifestyle behavior factors to visualize children’s weight status. Thus, sufficient social efforts are crucial for improving appropriate body image and lifestyle behaviors for children and adolescents.

## 5. Conclusions

Our study found that a discrepancy exists between children’s actual weight status and their perceived weight status. Girls were more likely than boys to overestimate their weight status. About 39.8% of obesity in boys could be accounted for by the interaction of their self-perceived weight status, their idealized male body image, and the consumption of meat. Additionally, protein food may affect the relationship between self-perceived weight status and obesity in Chinese children, especially in girls, and the relationship between self-perceived weight status and obesity may be influenced by sedentary behaviors in Chinese school-age girls. Therefore, schools and parents should take targeted measures to help children and adolescents improve their knowledge of healthy diets and increase their awareness of their actual weight status. This will contribute to improved awareness of weight status and healthy eating habits and, in turn, help Chinese children maintain a healthy body weight status.

## Figures and Tables

**Table 1:** Sample characteristics from the Childhood Obesity Study in China Mega-Cities during in 2015–2017.

	All (*n* = 3258)	Boys (*n* = 1651)	Girls (*n* = 1607)	*p*
Age (years)	12.2 ± 2.0	12.2 ± 1.9	11.2 ± 2.0	0.964
Body mass index (BMI) (kg/m^2^)	19.6 ± 3.8	20.1 ± 3.9	19.2 ± 3.6	<0.001
Diet (<3 d/week, %)				
Livestock meat	41.3	38.1	44.6	0.018
Poultry	66.9	62.8	71.1	0.001
Pure milk	28.8	25.8	31.9	0.005
Dairy products	51.8	53.4	50.2	0.012
Soybeans and soy products	71.5	69.1	74.0	0.053
Exercise & sedentary behaviors (<3 times/week, %)				
Watching TV/video/movies	80.0	78.3	81.8	<0.001
Go online/use a computer	76.5	75.1	77.9	0.676
Extracurricular physical activity	52.7	48.5	56.9	0.077
Demographics & family socioeconomic status				
Father’s BMI (kg/m^2^)	24.4 ± 3.7	24.4 ± 3.7	24.3 ± 3.7	0.159
Mather’s BMI (kg/m^2^)	22.3 ± 3.7	22.2 ± 3.7	22.4 ± 3.7	0.117
Parental highest education level (%)				0.184
Middle school or below	16.0	17.2	14.8	
High or vocational schools	28.2	28.9	27.6	
College or above	55.7	53.8	57.6	
Family homeownership (%)				0.791
Rent or share residency with relatives	26.8	27.2	26.3	
Own apartment	58.9	57.9	59.8	
Own house	12.6	12.7	11.8	

Values are means ± SDs unless otherwise indicated; P values were calculated by using *t*-test or chi-square test.

**Table 2:** Factor analysis of lifestyle behaviors (eating and physical activity behaviors) of Chinese school children from no repeat data in 2015–2017.

Variables	Factor 1	Factor 2	Factor 3	Factor 4
1. Livestock meat	···	0.781	···	···
2. Poultry meat	···	0.911	···	···
3. Pure milk	0.680	···	···	···
4. Dairy products	0.730	···	···	···
5. Soybeans and soy products	0.816	···	···	···
6. Watching TV/video/movies	···	···	0.780	···
7. Go online/use a computer	···	···	0.846	···
8. extracurricular physical activity	···	···	···	0.992
Characteristic root	1.780	1.600	1.380	1.016
Variance contribution rate (%)	22.24	20.00	17.25	12.69
Variance cumulative contribution rate (%)	22.24	42.24	59.50	72.19

Limited to four factors according to characteristic root. Factor load ≥0.65. ···, not reported.

**Table 3:** Associations of the interaction of self-assessed body weight status and lifestyle behaviors with obesity in Chinese school children in 2015–2017.

Self-Perceived Weight Status	Life Behavior Factors	All		Boys		Girls	
		Coef. (95%CI)	AP (%)	Coef. (95%CI)	AP (%)	Coef. (95%CI)	AP (%)
	Factor 1						
−	−	Reference		Reference		Reference	
−	+	0.44 (0.03, 0.84) *		0.69 (0.19, 1.20) **		−0.11 (−0.80, 0.58)	
+	−	3.23 (2.81, 3.65) ***		3.89 (3.30, 4.49) ***		2.44 (1.83, 3.04) ***	
+	+	3.55 (3.12, 3.98) ***	26.2 *	3.67 (3.09, 4.25) ***	−26.9	3.08 (2.47, 3.70) ***	48.2 *
	Factor 2						
−	−	Reference		Reference		Reference	
−	+	−0.14 (−0.55, 0.26)		−0.12 (−0.62, 0.37)		−0.12 (−0.83, 0.58)	
+	−	2.95 (2.56, 3.35) ***		3.07 (2.52, 3.62) ***		2.72 (2.15, 3.29) ***	
+	+	3.19 (2.79, 3.60) ***	21.8	3.57 (3.00, 4.15) ***	39.8 *	2.80 (2.20, 3.40) ***	7.6
	Factor 3						
−	−	Reference		Reference		Reference	
−	+	0.07 (−0.34, 0.48)		0.00 (−0.50, 0.50)		0.22 (−0.49, 0.93)	
+	−	3.07 (2.68, 3.46) ***		3.44 (2.90, 3.99) ***		2.70 (2.10, 3.29) ***	
+	+	3.29 (2.88, 3.69) ***	19.1	3.34 (2.80, 3.89) ***	−10.8	3.13 (2.52, 3.75) ***	34.6 *
	Factor 4						
−	−	Reference		Reference		Reference	
−	+	0.36 (−0.06, 0.77)		0.11 (−0.38, 0.61)		0.92 (0.16, 1.68) *	
+	−	3.25 (2.82, 3.68) ***		3.36 (2.80, 3.93) ***		3.26 (2.53, 3.98) ***	
+	+	3.43 (3.01, 3.87) ***	15.7	3.56 (2.98, 4.14) ***	17.6	3.43 (2.71, 4.15) ***	11.4

* <0.05, ** <0.01, *** <0.001. Regression coefficients (95% CIs) were estimated with mixed-effects regression models with different levels of adjustment. Adjusted for age, gender. Subgroup analysis applied same mixed model except for stratified variable. Self-perceived weight status: “−” non-overweight, “+” overweight. The four factors are stratified by the P_50_ of their own factor score. Factor 1: “−” Not eat protein foods regularly, “+” Eat protein foods regularly. Factor 2: “−” Not eat meat regularly, “+” Eat meat regularly. Factor 3: “−” Not use sedentary behaviors regularly, “+” Use sedentary behaviors regularly. Factor 4: “−” Infrequent extracurricular physical activity, “+” Frequent extracurricular physical activity.

## Data Availability

The data presented in this study are available on request from the corresponding author.

## Appendix A

**Table A1:** Age- and gender-specific BMI cut-off points for overweight and obesity.

Age (Year)	Boys		Girls	
	Overweight	Obesity	Overweight	Obesity
6.0~	16.4	17.7	16.2	17.5
6.5~	16.7	18.1	16.5	18.0
7.0~	17.0	18.7	16.8	18.5
7.5~	17.4	19.2	17.2	19.0
8.0~	17.8	19.7	17.6	19.4
8.5~	18.1	20.3	18.1	19.9
9.0~	18.5	20.8	18.5	20.4
9.5~	18.9	21.4	19.0	21.0
10.0~	19.2	21.9	19.5	21.5
10.5~	19.6	22.5	20.0	22.1
11.0~	19.9	23.0	20.5	22.7
11.5~	20.3	23.6	21.1	23.3
12.0~	20.7	24.1	21.5	23.9
12.5~	21.0	24.7	21.9	24.5
13.0~	21.4	25.2	22.2	25.0
13.5~	21.9	25.7	22.6	25.6
14.0~	22.3	26.1	22.8	25.9
14.5~	22.6	26.4	23.0	26.3
15.0~	22.9	26.6	23.2	26.6
15.5~	23.1	26.9	23.4	26.9
16.0~	23.3	27.1	23.6	27.1
16.5~	23.5	27.4	23.7	27.4
17.0~	23.7	27.6	23.8	27.6
17.5~	23.8	27.8	23.9	27.8
18.0~	24.0	28.0	24.0	28.0

**Table A2:** Additive interactions result by Andersson’s Excel (Only show the positive results in Table 3).

A: The Interactions between Self-Perceived Weight Status and Factor 1			
	Factor 1	Self-Perceived Weight Status	Factor 1& Self-Perceived Weight Status
Regr.(regression) Coefficients	0.43594	3.22908	3.55456
Cov Factor 1	0.0435	0.02584	0.02591
Cov Self-perceived weight status	0.02584	0.04537	0.031
Cov Factor 1 & self-perceived weight status	0.02591	0.031	0.0481
Exposure	RR	Lower	Upper
Factor 1	1.546	1.028	2.327
Self-perceived weight status	25.256	16.636	38.343
Factor 1 & self-perceived weight status	34.972	22.753	53.754
Measure	Estimate	Lower	Upper
RERI	9.17	−1.975	20.314
AP	0.262	0.008	0.517
S	1.37	0.959	1.957
**B: the interactions between self-perceived weight status and factor 1 in girls**			
	Factor 1	Self-perceived weight status	Factor 1 & self-perceived weight status
Regr.(regression) Coefficients	−0.11338	2.43583	3.08345
Cov Factor 1	0.12427	0.06297	0.06294
Cov Self-perceived weight status	0.06297	0.09515	0.07071
Cov Factor 1 & self-perceived weight status	0.06294	0.07071	0.09761
Exposure	RR	Lower	Upper
Factor 1	0.893	0.447	1.782
Self-perceived weight status	11.425	6.242	20.914
Factor 1 & self-perceived weight status	21.834	11.835	40.278
Measure	Estimate	Lower	Upper
RERI	10.516	1.197	19.834
AP	0.482	0.248	0.715
S	2.019	1.243	3.281
**C: the interactions between self-perceived weight status and factor 2 in boys**			
	Factor 2	Self-perceived weight status	Factor 2 & self-perceived weight status
Regr.(regression) Coefficients	−0.12473	3.0694	3.57187
Cov Factor 2	0.06305	0.03385	0.03442
Cov Self-perceived weight status	0.03385	0.07989	0.037
Cov Factor 2 & self-perceived weight status	0.03442	0.037	0.08549
Exposure	RR	Lower	Upper
Factor 2	0.883	0.54	1.444
Self-perceived weight status	21.529	12.372	37.464
Factor 2 & self-perceived weight status	35.583	20.062	63.113
Measure	Estimate	Lower	Upper
RERI	14.171	-4.189	32.531
AP	0.398	0.041	0.756
S	1.694	0.914	3.141
**D: the interactions between self-perceived weight status and factor 3 in girls**			
	Factor 3	Self-perceived weight status	Factor 4 & self-perceived weight status
Regr.(regression) Coefficients	0.22064	2.69594	3.13756
Cov Factor 3	0.13052	0.05985	0.06333
Cov Self-perceived weight status	0.05985	0.09131	0.06727
Cov Factor 3 & self-perceived weight status	0.06333	0.06727	0.09778
Exposure	RR	Lower	Upper
Factor 3	1.247	0.614	2.531
Self-perceived weight status	14.819	8.196	26.795
Factor 3 & self-perceived weight status	23.048	12.487	42.541
Measure	Estimate	Lower	Upper
RERI	7.981	−1.783	17.745
AP	0.346	0.049	0.644
S	1.567	0.968	2.538
